# The Fischer 344 Rat Reflects Human Susceptibility to *Francisella* Pulmonary Challenge and Provides a New Platform for Virulence and Protection Studies

**DOI:** 10.1371/journal.pone.0009952

**Published:** 2010-04-01

**Authors:** Heather J. Ray, Ping Chu, Terry H. Wu, C. Rick Lyons, Ashlesh K. Murthy, M. Neal Guentzel, Karl E. Klose, Bernard P. Arulanandam

**Affiliations:** 1 South Texas Center for Emerging Infectious Diseases and Department of Biology, University of Texas at San Antonio, San Antonio, Texas, United States of America; 2 Center for Infectious Disease and Immunity, Department of Internal Medicine, The University of New Mexico Health Science Center, Albuquerque, New Mexico, United States of America; Duke University, United States of America

## Abstract

**Background:**

The pathogenesis of *Francisella tularensis*, the causative agent of tularemia, has been primarily characterized in mice. However, the high degree of sensitivity of mice to bacterial challenge, especially with the human virulent strains of *F. tularensis*, limits this animal model for screening of defined attenuated vaccine candidates for protection studies.

**Methods and Findings:**

We analyzed the susceptibility of the Fischer 344 rat to pulmonary (intratracheal) challenge with three different subspecies (subsp) of *F. tularensis* that reflect different levels of virulence in humans, and characterized the bacterial replication profile in rat bone marrow-derived macrophages (BMDM). In contrast to the mouse, Fischer 344 rats exhibit a broader range of sensitivity to pulmonary challenge with the human virulent subsp. *tularensis* and *holarctica*. Unlike mice, Fischer rats exhibited a high degree of resistance to pulmonary challenge with LVS (an attenuated derivative of subsp. *holarctica*) and subsp. *novicida*. Within BMDM, subsp. *tularensis* and LVS showed minimal replication, subsp. *novicida* showed marginal replication, and subsp. *holartica* replicated robustly. The limited intramacrophage replication of subsp. *tularensis and novicida* strains was correlated with the induction of nitric oxide production. Importantly, Fischer 344 rats that survived pulmonary infection with subsp. *novicida* were markedly protected against subsequent pulmonary challenge with subsp. *tularensis*, suggesting that subsp. *novicida* may be a useful platform for the development of vaccines against subsp. *tularensis*.

**Conclusions:**

The Fischer 344 rat exhibits similar sensitivity to *F. tularensis* strains as that reported for humans, and thus the Fischer 344 ray may serve as a better animal model for tularemia vaccine development.

## Introduction

*Francisella tularensis* is a gram negative bacterium that is the causative agent of the human disease tularemia [1], [2]. There is currently no licensed vaccine for human use, but extensive efforts are underway to identify potential vaccine candidates for this biothreat agent. Most of our current understanding of pathogenesis and host immune responses comes from studies using the mouse model of *F. tularensis* infection [3], [4], [5], [6], [7]. While the murine tularemia model has extended our understanding of the disease process, the extreme sensitivity of the mouse to *F. tularensis* challenge limits the usefulness of this model in protection studies.

*F. tularensis* subsp. *tularensis* and *holartica* are virulent in humans [1], [8] and mice (LD_50_ <10 CFU for subsp. *tularensis* and *holartica* in mice via the pulmonary route) [9], [10]. In contrast, *F. tularensis* subsp. *novicida* and LVS, an attenuated vaccine strain derived from subsp. *holarctica*, are avirulent in humans, [1], [10] but highly virulent in mice (LD_50_ <10 CFU for subsp. *novicida* and LD_50_ <10^4^ CFU for LVS in mice via the pulmonary route) [11], [12], [13]. Various vaccination studies, using either LVS [6], [9], [14] or a variety of defined *F. tularensis* vaccine strains [10], [12], [15], have been able to show some level of protective efficacy in mice, but only against very low challenge doses (10–500 CFU of subsp. *tularensis*) [10], [16], [17]. Thus, it would be highly beneficial to identify an alternative animal model that exhibits similar sensitivities to *Francisella* infection as humans and provides a more reflective platform for assessing vaccine candidates, with a wider window of titratable protection.

One such alternative animal model for *F. tularensis* infections is the rat. Historical studies suggested that “white rats” were much less susceptible to *F. tularensis* challenges than mice [18], [19]. It also was found that several rat strains, including the Fischer 344 rat, were resistant to LVS challenge [20], [21], [22]. However, after these initial studies, the rat model was overlooked in favor of the mouse model, due in part to the availability of reagents and knockout/transgenic strains to aid in understanding the mechanisms of host immunity. Two recent studies have stimulated renewed interest in the rat model of *Francisella* infection. Conlan and colleagues [23] compared the relative susceptibilities of Fischer 344 and Sprague-Dawley rats to intraperitoneal (IP) *F. tularensis* subsp. *tularensis* and *holarctica* challenges, and suggested that the decreased sensitivity of the Sprague-Dawley rats to these strains may provide a model to assess host innate immune defenses. Also, Wu *et. al* [24] have reported on the efficacy of subcutaneous or intradermal LVS vaccination in Fischer 344 rats, which affords protection against a broader range of subsp. *tularensis* pulmonary challenges.

In the current study, we sought to further extend these observations with a comprehensive analysis of pulmonary challenge of Fischer 344 rats with three different subsps. of *F. tularensis* that exhibit varying degrees of virulence in humans. We further characterized the bacterial replication profile of these strains within rat macrophages. Our studies suggest that the response of the Fischer 344 rat to the various *F. tularensis* strains may be more reflective of how humans respond to these organisms. These studies provide additional insight into the utility of the rat model for screening potential vaccine candidates against *F. tularensis*.

## Results

### *In vivo* susceptibility of Fischer 344 rats to pulmonary challenge with *Francisella* strains

Recent publications have characterized the susceptibility of Fischer 344 rats to LVS and *F. tularensis* subsp. *tularensis* pulmonary challenge [24] and to IP infections with *F. tularensis* subsp. *holarctica* (both wildtype and LVS strains) and *F. tularensis* subsp. *tularensis* [23]. However, there has yet to be a full comparison of Fischer 344 rats infected with the four most widely studied strains via the same route of inoculation. Since the pulmonary route of infection is the most relevant for human vaccine development, we performed experiments to determine the LD_50_ of four *F. tularensis* strains, *F. tularensis* subsp. *tularensis* SCHU S4, *F. tularensis* subsp. *holarctica* OR960246, *F. tularensis* subsp. *holarctica* LVS and *F. tularensis* subsp. *novicida* U112, via pulmonary challenge of Fischer 344 rats.

Groups (n = 6) of rats were inoculated by intratracheal instillation with increasing doses (10^2^ to 10^7^ CFU) of subsp. *novicida*, LVS, subsp. *holarctica* and subsp. *tularensis* and monitored daily for survival. As shown in Table 1, rats were highly resistant to subsp. *novicida* challenge, with an approximate LD_50_ of 5×10^6^ CFU. As recently reported by Wu *et. al*. [24], rats were extremely resistant to LVS challenge. In fact, the rats in our study did not exhibit any overt signs of illness with doses of LVS as high as 10^7^ CFU. In contrast, rats were more sensitive to subsp. *holarctica* challenge (approx. LD_50_ of 1×10^5^ CFU). Rats showed the highest sensitivity to pulmonary infection with subsp. *tularensis* (approx. LD_50_ of 5×10^2^ CFU). Humans also show the greatest susceptibility to subsp. *tularensis* infection, followed by susceptibility to subsp. *holarctica* infection. Humans also are highly resistant to LVS infections (since this was developed as a potential human vaccine), as well as to subsp. *novicida* infections. While some rats did succumb to pulmonary challenge with this subsp., it was only with very high doses. The early time to death observed in those rats which did succumb, along with the increased stimulatory properties of subsp. *novicida* LPS over that of other subsp. [25], suggests that death may have been caused by toxicity from the large number of organisms rather than by a productive infection. Overall, this data suggests that *F. tularensis* infections of the Fischer 344 rat may be a more representative model of human pulmonary tularemia than the widely used mouse model.

**Table 1:** Intratracheal LD_50_ Doses of *Francisella* Strains in Fischer 344 Rats.

Bacterial Strain	Intratracheal LD_50_ Dose (Approximate)	MTD
subsp. *novicida*	5×10^6^ CFU	3 days
LVS	>1×10^7^ CFU	≫30 days
subsp. *holarctica*	1×10^5^ CFU	10 days
subsp. *tularensis*	5×10^2^ CFU	10 days

Groups of Fischer 344 rats (n = 6) were challenged intratracheally with increasing doses (10^2^ to 10^7^ CFU) of subsp. *novicida* strain U112, subsp. *holarctica* strain LVS, subsp. *holarctica* strain OR96-0246, or subsp. *tularensis* strain SCHU S4 and monitored daily for morbidity and mortality and the LD_50_ and mean time to death (MTD) calculated. Results for each strain are representative of at least two separate experiments.

### Generation of bone marrow derived rat macrophages and analysis of phagocytic ability

Because *F. tularensis* can survive and replicate within macrophages, a key attribute to its virulence, mouse bone marrow derived macrophages (BMDM) have been used extensively as an *in vitro* model to study *F. tularensis* infections. In order to further characterize the Fischer 344 rat model, we determined how the various *F. tularensis* strains replicated in rat BMDM. We first compared the basal phagocytic capability of BMDM generated from Fisher 344 rats to that of BMDM derived from BALB/c mice to ensure that the macrophages generated from the rat were functionally similar to those from mice.

**Figure 1:**
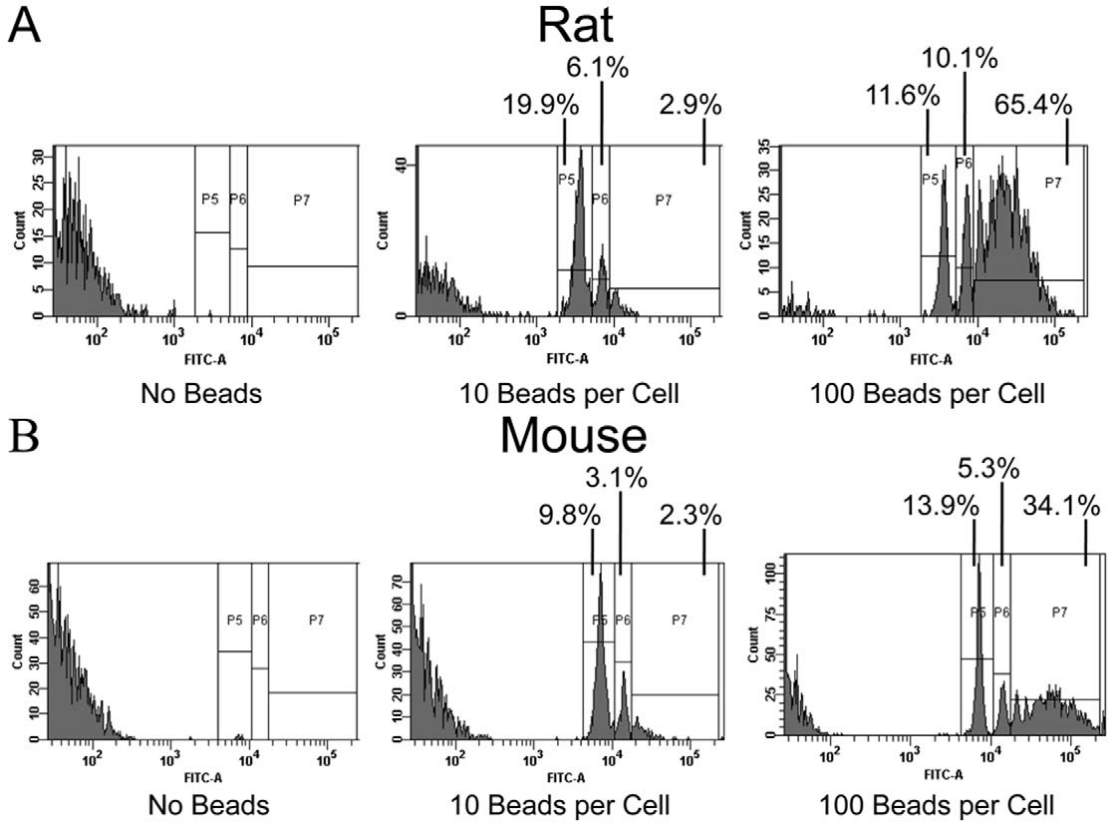
Comparison of basal phagocytic ability of BMDM from Fischer 344 rats and BALB/c mice. Bone marrow derived macrophages from (A) Fischer 344 rats and (B) BALB/c mice were seeded in 6 well plates (1×10^6^ cells/well) and allowed to adhere. Cells were incubated with fluorescent microspheres (10 or 100 beads/cell) for 1 hr to allow for phagocytosis. Cells were collected into 5 ml polystyrene tubes and stained with either mouse anti-rat CD11b AF647 or rat anti-mouse CD11b APC to confirm the macrophage population. The numbers of intracelllular beads were quantified by flow cytometry (P5 = 1 bead, P6 = 2 beads, P7≥3 beads). Results are representative of two separate experiments.

To compare gross phagocytic ability, macrophages were seeded in 6 well tissue culture plates (1×10^6^ cells/well) and incubated for two hr with fluorescently labeled microbeads at a concentration of either 10 or 100 beads per cell. Extracellular beads were then washed off and the cells were labeled with either CD11b-AlexaFluor 647 (Fisher 344 BMDM) or CD11b-APC (BALB/c BMDM) to confirm the macrophage population. Cells were then analyzed for the presence of intracellular beads by flow cytometry. In a histogram plot (Fig. 1), the presence of one bead per cell is indicated by the first positive peak (gate P5), and increasing numbers of beads (2 beads and ≥3 beads) are evidenced by successive peaks of increasing fluorescence intensity (gates P6 and P7, respectively). As shown in Figure 1, at an initial concentration of 10 beads per cell, approximately 29% of rat and 15% of mouse BMDM contained beads, with the majority of cells containing only one bead. When the concentration of beads was increased to 100 beads per cell, a greater percentage of both cell types (87% of rat and 49% of mouse BMDM) contained beads, with the greatest number of cells having taken up 3 or more beads. These results indicated that BMDM generated from Fischer 344 rats have a basal phagocytic ability better than those from BALB/c mice.

### Uptake and replication of *F. tularensis* strains within Fischer 344 bone marrow derived macrophages

**Figure 2:**
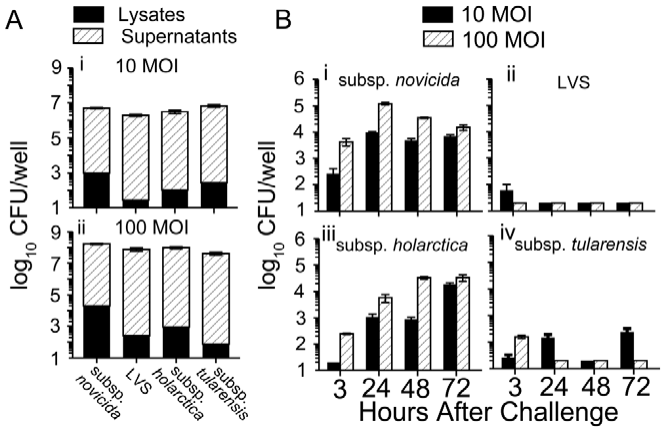
Uptake and replication of *Francisella* strains within Fischer 344 rat BMDM. Bone marrow derived macrophages from Fischer 344 rats were seeded in 96 well plates (2×10^5^ cells/well) and allowed to adhere. Cells were infected (10 or 100 MOI) for two hr with subsp. *novicida*, LVS, subsp. *holarctica*, or subsp. *tularensis*. (A) Serial dilutions of culture supernatants (extracellular bacteria) and cell lysates (intracellular bacteria) at (i) 10 MOI and (ii) 100 MOI were plated to determine ability of macrophages to take up bacteria. Results are representative of two separate experiments. (B) Following a 2 hr infection period, cells were treated with gentamicin for 1 hr, and then incubated at 37°C for 72 hr. At the indicated time points (3, 24, 48 and 72 h), cells were lysed and serial dilutions of lysates were plated to quantify intracellular bacteria. Intracellular replication profiles of (i) subsp. *novicida*, (ii) LVS, (iii) subsp. *holarctica*, or (iv) subsp. *tularensis*. Results are representative of at least three separate experiments.

Once we determined that BMDM from Fischer 344 rats readily phagocytosed inert beads, we analyzed the ability of these cells to phagocytose *F. tularensis* strains. Rat BMDM were seeded into 96 well plates (2×10^5^ cells/well), allowed to adhere, and then infected (10 and 100 MOI) with subsp. *novicida*, LVS, subsp. *holarctica* and subsp. *tularensis* for two hr to allow phagocytosis of bacteria. Immediately following this infection period, supernatants were removed, cells were lysed with 0.2% deoxycholate, and serial dilutions of both lysates and supernatants were plated to determine CFU. As shown in Figure 2A, there were differences in bacterial numbers of the various strains in the cell lysates, which represent intracellular bacteria. The Fischer 344 BMDM were able to phagocytose all of the four tested strains, but phagocytosis of subsp. *novicida* was notably higher (1–2 log_10_) than phagocytosis of LVS, subsp. *holarctica* and subsp. *tularensis*.

Since rat BMDM were capable of phagocytosing all of the *F. tularensis* strains, we also analyzed the replication kinetics of these strains within the cells. Rat BMDM were seeded in 96 well plates, allowed to adhere, and infected with subsp. *novicida*, LVS, subsp. *holarctica* and subsp. *tularensis* for two hr, as above. Cells were then washed, treated for one hr with gentamicin to kill extracellular bacteria, and then incubated for up to 72 hr. At the indicated time points (3, 24, 48, 72 h) after infection, supernatants were removed, cells were lysed and serial dilutions of lysates were plated to determine CFU, which represent intracellular bacteria. As seen in Figure 2B(i), 2 to 4 log_10_ of subsp. *novicida* were present intracellularly at 3 hr after infection with 10 and 100 MOI, as seen previously. By 24 h, the bacteria had replicated minimally (approximately 1.5 log_10_), after which the intracellular bacterial counts were reduced slightly, out to 72 hr. As shown in Figure 2B(ii), very few (<2 log_10_) LVS were present intracellularly at 3 h after infection, and intracellular bacteria remained at or below the level of detection throughout the 72 hr time course. In contrast, subsp. *holarctica* (Figure 2B(iii)) increased from low intracellular numbers (1–3 log_10_) at 3 h after infection throughout the entire time period, with an overall replication of 3 to 4 log_10_ by 72 hr after infection. Subsp. *tularensis* (Figure 2B(iv)) was found intracellularly in similar numbers to subsp. *holarctica* at 3 hr, but failed to replicate over the time course of the experiment, with few intracellular bacteria found between 24 and 72 hr.

**Figure 3:**
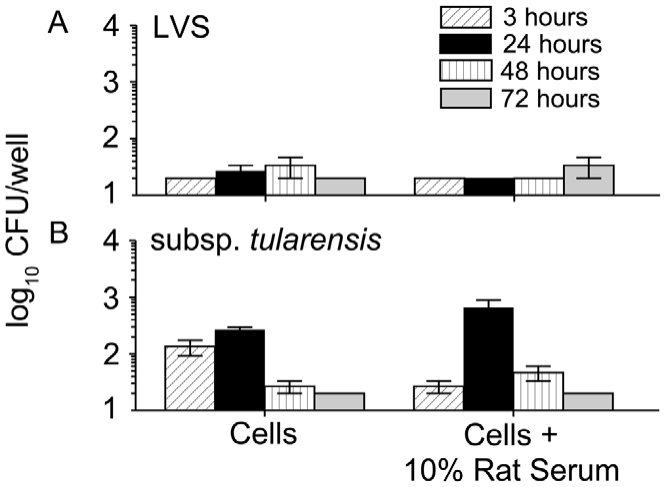
Effect of naïve rat serum on uptake and replication of *Francisella* strains within Fischer 344 rat BMDM. Bone marrow derived macrophages were seeded in 96 well plates (2×10^5^ cells/well) and allowed to adhere. Cells were infected (10 MOI) with (A) LVS or (B) subsp. *tularensis* for 2 hr with or without 10% naïve rat serum as an opsonin, treated for 1 hr with gentamicin, and incubated at 37°C for 72 hr. At the indicated time points (3, 24, 48, or 72 h) cells were lysed and serial dilutions of lysates were plated to quantify intracellular bacteria. Results are representative of two separate experiments.

It has been previously shown that the addition of homologous serum facilitates greater uptake of *F. tularensis* into human monocytes [26], [27]. Since LVS and subsp. *tularensis* exhibited the lowest initial uptake into rat BMDM, we investigated whether the addition of homologous rat serum would increase the initial uptake, as well as the intracellular replication of the organisms. Prior to inoculation into the BMDM, the LVS and subsp. *tularensis* strains were first added to media which included 10% (v/v) naïve Fischer 344 rat serum and placed in a shaking incubator (37°C, 220 RPM) for 30 minutes to promote opsonization. Bacteria (10 MOI) were then added to the rat BMDM for 2 hr, gentamicin treated, and intracellular numbers analyzed as above. As seen in Figure 3, the addition of homologous serum did not increase uptake or the replication of either LVS (3A) or subsp. *tularensis* (3B).

### Nitric oxide production by bone marrow derived macrophages infected with *F. tularensis* strains

**Figure 4:**
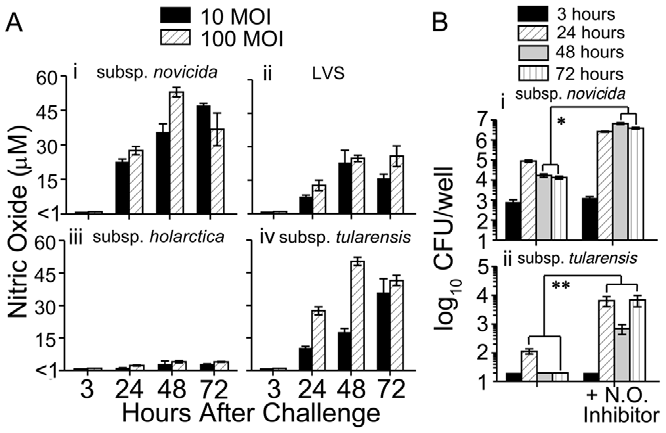
Contribution of Fischer 344 rat BMDM nitric oxide production on intracellular replication of *Francisella* strains. (A) Supernatants from intracellular replication assays (3, 24, 48 and 72 h) were analyzed for the presence of nitric oxide. Concentrations of nitric oxide following infection of BMDM with (i) subsp. *novicida*, (ii) LVS, (iii) subsp. *holarctica*, and (iv) subsp. *tularensis*. Results are representative of three separate experiments. (B) Fischer 344 BMDM were seeded in 96 well plates (2×10^5^ cells/well) and allowed to adhere. Cells were infected with 10 MOI of (i) subsp. *novicida* or (ii) subsp. *tularensis* for 2 hr, with or without N-methyl-L-arginine acetate salt as a nitric oxide inhibitor, treated for 1 hr with gentamicin, and incubated at 37°C for 72 hr. At the indicated time points (3, 24, 48 and 72 h) after challenge, cells were lysed and serial dilutions were plated to quantify intracellular bacteria. Results are representative of two separate experiments. * *P*<0.001, ** *P*<0.05.

In order to investigate the possible basis for differences in intracellular replication between the *F. tularensis* strains, we analyzed the culture supernatants from intramacrophage replication assays for the presence of inflammatory mediators, such as nitric oxide and TNFα, which are known to be produced by macrophages after infection [28]. Induction of nitric oxide in infected mouse macrophages has previously been shown to control *F. tularensis* replication [4], [29], [30], [31], thus we investigated whether nitric oxide was produced by Fischer 344 BMDM upon infection with *F. tularensis* using a spectrophotometric assay with Greiss reagent. As shown in Figure 4A, nitric oxide was not detectable at 3 hr after infection of BMDM with any strain. Macrophages infected with subsp. *holarctica* (4A(iii)) did not induce significant levels of nitric oxide, which correlates with the high degree of intracellular replication exhibited by this strain. In contrast, macrophages infected with subsp. *tularensis* (4A(iv)) and LVS (4A(ii)) expressed relatively high levels of nitric oxide and these strains exhibited minimal replication in these cells. Thus, the comparative levels of nitric oxide produced by the infected macrophages correlated with intracellular replication of the individual *F. tularensis* strains. However, macrophages infected with subsp. *novicida* (4A(i)) induced the greatest production of nitric oxide, with up to 50 µM present at 48 hr after challenge, despite the ability of subsp. *novicida* to replicate in these cells. Still, subsp. *novicida* only replicated minimally in the macrophages, suggesting that nitric oxide may contribute to limiting intracellular replication of this strain as well. Levels of TNFα in culture supernatants of infected macrophages were also measured and it was found that only macrophages infected with subsp. *novicida* exhibited significant levels of this cytokine (data not shown), demonstrating a lack of correlation of rat BMDM TNFα production and the ability of the individual *F. tularensis* strains to replicate intracellularly.

### Inhibition of nitric oxide production leads to increased intracellular *F. tularensis* replication

Since the *F. tularensis* strains that either replicated minimally or failed to replicate within rat BMDM (subsp. *tularensis*, LVS and subsp. *novicida*) also induced high levels of nitric oxide within the BMDM, we determined the effect that inhibition of nitric oxide production by the macrophages would have on *F. tularensis* replication. BMDM were seeded as above and infected with 10 MOI of subsp. *novicida* or subsp. *tularensis*, the two strains which induced the greatest levels of nitric oxide, and nitric oxide inhibitor (1 mM concentration of N-methyl-L-arginine acetate salt), was added to infected macrophages. As shown in figure 4B, addition of nitric oxide inhibitor to the macrophages led to an increase in intracellular replication of 2–3 logs for both subsp. *novicida* (4B(i)) and subsp. *tularensis* (4B(ii)) when compared to infected macrophages without inhibitor added. This demonstrates that nitric oxide production by the Fischer 344 macrophages is involved in limiting bacterial replication of these two *F. tularensis* strains.

### Ability of *F. tularensis* strains to replicate within Fischer 344 rat hepatocytes

**Figure 5:**
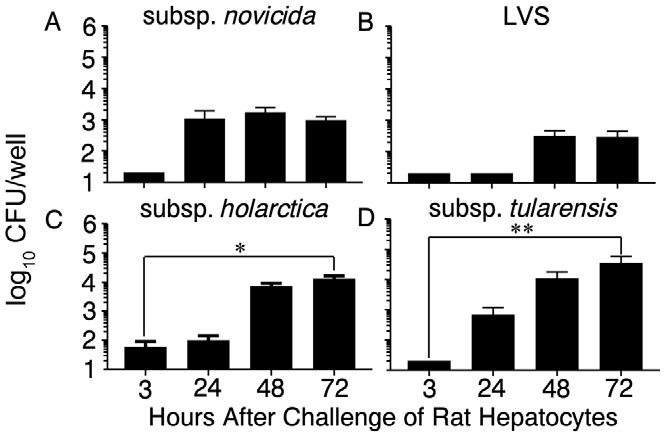
Intracellular replication of *Francisella* strains within Fischer 344 hepatocytes. Livers were collected from Fischer 344 rats and hepatocytes were isolated. Cells were seeded in 96 well plates (2×10^5^ cells/well) and allowed to adhere. Cells were infected with 10 MOI of (A) subsp. *novicida*, (B) LVS, (C) subsp. *holarctica* or (D) subsp. *tularensis* for 2 hr, gentamicin treated for 1 hr, and incubated at 37°C for 72 hr. At the indicated time points after infection (3, 24, 48 and 72 hr), cells were lysed and serial dilutions were plated to quantify intracellular bacteria. Results are representative of three separate experiments. *P = 0.02, **P<0.01.

The failure of subsp. *tularensis* to replicate within rat BMDM, despite its high virulence within the rat, suggests that there might be other cell types that are more permissive for intracellular subsp. *tularensis* replication. It has been reported that following pulmonary challenge with subsp. *tularensis*, the bacteria are disseminated to the livers of Fischer 344 rats [24]. Since *F. tularensis* is able to replicate *in vitro* within mouse hepatocytes [31], we decided to examine *F. tularensis* replication within rat hepatocytes. Primary hepatocytes isolated from the livers of Fischer 344 rats were infected with 10 MOI of subsp. *novicida*, LVS, subsp. *holarctica* and subsp. *tularensis*, and intracellular replication for each strain was determined. As shown in Figure 5A, subsp. *novicida* exhibited a similar replicative profile within primary hepatocytes (an intial replication of 1–2 log_10_ at 24 hr after which intracellular numbers remained steady through 72 hr) as that seen with BMDM (Fig. 2B(i)), except that much fewer bacteria were found intracellularly at 3 hr in hepatocytes than in BMDM. Both LVS (Fig. 5B), that was unable to replicate significantly, and subsp. *holarctica* (Fig. 5C) that had relatively few bacteria intracellularly at 3 hr post infection but showed continual replication throughout the 72 hr time period (3–4 log_10_ overall replication), exhibited similar replication kinetics to what was seen in BMDM (Fig. 2B(ii) and Fig. 2B(iii) respectively). Subsp. *tularensis* was also found at relatively low levels intracelullarly at 3 hr post infection (Fig. 5D), however, in contrast to BMDM (Fig. 2B(iv)), subsp. *tularensis* demonstrated significant (*P*<0.01) intracellular replication in rat hepatocytes throughout the 72 hr time course (3–4 logs replication). Thus, while subsp. *novicida* exhibited decreased intracellular numbers in hepatocytes, the human virulent subsp. *holarctica* was equally able to replicate in both cell types and the most virulent subsp. *tularensis* strain, which was unable to replicate within rat BMDM, was competent for replication within rat hepatocytes, suggesting that cell types other than macrophages may be important for replication of this pathogen in the rat.

### Ability of subsp. *novicida* to induce a protective immune response against subsp. *tularensis* in the Fischer 344 rat

**Figure 6:**
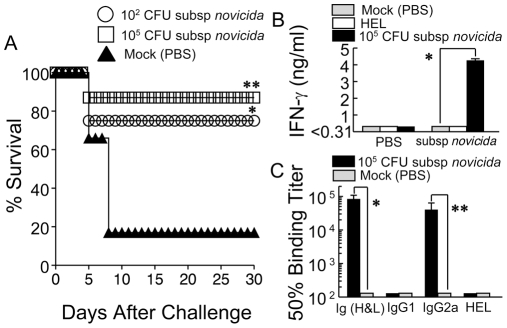
Protective efficacy of subsp. *novicida* vaccination against subsequent subsp. *tularensis* challenge in Fischer 344 rats. Groups of Fischer 344 rats (n = 6) were vaccinated intratracheally with 10^2^ or 10^5^ CFU of subsp. *novicida* in PBS or mock vaccinated with PBS alone. (A) Thirty days later, rats were challenged intratracheally with subsp. *tularensis* (10^4^ CFU) and monitored daily for morbidity and mortality. *P = 0.182, **P<0.05 (B) Fourteen days after vaccination with 10^5^ CFU of subsp. *novicida* or PBS alone, rats were sacrificed, cervical lymph nodes removed, and whole cell populations were recalled with 10^5^ CFU of UV-inactivated subsp. *novicida*, media alone, or the unrelated antigen HEL for 72 hr. Culture supernatants were analyzed for antigen-specific IFN-γ production. *P<0.001 (C) Thirty days after vaccination with 10^5^ CFU of subsp. *novicida* or PBS alone, blood was collected and sera were prepared. Sera were analyzed for antigen-specific total antibody (Ig H & L), IgG1, and IgG2a, as well as reaction to the unrelated antigen HEL by ELISA. Results are represented as 50% binding titers. Results are representative of two separate experiments. *P<0.01, **P = 0.118.

Our data indicate that the sensitivity of the Fischer 344 rat to the different *F. tularensis* strains is more similar to human sensitivity to these strains, than is that of the mouse. Thus, *F. tularensis* infections in the Fischer 344 rat may be more representative of human pulmonary tularemia than the mouse model. In particular, the high level of resistance of the rat (LD_50_ ∼10^6^ CFU) to pulmonary subsp. *novicida* challenges compared to the exquisite sensitivity of the mouse to this same challenge (LD_50_ ∼10 CFU) is striking, considering that subsp. *novicida* has extremely low virulence for humans (avirulent for immunocompetent individuals). Given the (1) high degree of resistance of the rats to pulmonary subsp. *novicida* challenge, (2) the genetic similarity (98.1% homology between sequences common to U112 and SCHU S4 [7], [32]) with subsp. *tularensis* and (3) similar growth characteristics in human macrophages [33], we determined whether animals exposed to subsp. *novicida* would be protected against subsequent challenge with the type A strain. Rats challenged intratracheally (IT) with subsp. *novicida* were rested for 30 days and subsequently challenged IT with subsp. *tularensis* (10^4^ CFU). As shown in Fig. 6A, rats i.t. challenged with subsp. *novicida* (10^2^ and 10^5^ CFU respectively) were greatly protected (*P* = 0.182 and *P*<0.05, respectively) following a lethal pulmonary subsp. *tularensis* challenge (75% and 87% survival, respectively). In contrast, the majority of rats not exposed to subsp. *novicida* (mock vaccinated) but challenged with the same dose of subsp. *tularensis*, succumbed to this infection (17% survival). These data demonstrate protective efficacy of a live subsp. *novicida* vaccination in rats against subsequent subsp. *tularensis* challenge via the pulmonary route.

We analyzed the cellular and humoral immune profiles induced in rats by IT subsp. *novicida* inoculation. Rats challenged IT (10^5^ CFU) with subsp. *novicida* induced significant (*P*<0.001) amounts of antigen-specific IFN-γ production (4.26 ng/ml) in isolated cervical lymph node cells compared to the same cells isolated from mock-challenged animals (Fig. 6B), or rat cervical lymph node cells cultured with the unrelated antigen hen egg lysozyme (HEL). Serum antibody profiles were determined 30 days following IT subsp. *novicida* challenge. Strikingly, rats exposed to subsp. *novicida* via the intratracheal route (10^5^ CFU) showed robust total antibody production (84065.78±24337) and exhibited a polarized (Th1) IgG2a response (40281.7±23519) (Fig. 6C). These results collectively demonstrate for the first time, using the rat model, that IT vaccination with live subsp. *novicida* induces a potent cellular and humoral response and confers significant protection to subsequent pulmonary challenge with subsp. *tularensis*.

## Discussion

Macrophages are important host cells for intracellular *F. tularensis* replication [4], [7], [27]. In mouse BMDM, the type A, type B, LVS and subsp. *novicida* strains replicate to 3–4 log_10_ within 24 h of challenge [7], [13], [34]. In contrast, while subsp. *novicida* is considered avirulent in humans, the bacterium was found to replicate 3–4 log_10_ by 72 h of culture in human peripheral blood monocytes and monocyte derived macrophages (hMDM) [33]. LVS and subsp. *tularensis* also have been shown to replicate in hMDMs, however opsonization with autologous serum is required for efficient uptake and replication of these two strains [26], [27], [35]. In the rat, we found that LVS was unable to replicate in Fischer 344 BMDM, and that while subsp. *novicida* was phagocytosed to a greater extent initially, replication was limited to 1–2 log_10_ by 24 h. Subsp. *tularensis* and *holarctica* were taken up in relatively low numbers initially, followed by minimal replication with subsp. *tularensis*, but continued replication (2–3 log_10_) with subsp. *holarctica*. Therefore, while absolute numbers of subsp. *novicida* and subsp. *holarctica* recovered were similar (approx. 10^5^ CFU), subsp. *holarctica* was able to replicate to a much greater degree. These differences in intramacrophage replication may not be attributed to a defect in uptake, since Fischer 344 rat BMDM exhibited better basal phagocytic ability to mouse BMDM. Moreover, inclusion of homologous rat serum did not facilitate increased uptake or replication of LVS or subsp. *tularensis* in rat BMDM, in contrast to reports with human macrophages. One of the primary differences between human and rat macrophage is the capacity to produce NO *in vitro* [36], [37]. Whereas human macrophages produce minimal NO upon LPS stimulation, rat macrophages have been shown to express 5-fold greater production of NO than mouse cells [38]. To this end, we found that subsp. *novicida* and subsp. *tularensis* induced the production of NO, and that addition of a NO inhibitor during the culture period increased the overall replication of both strains by 3 log_10_ at 72 h. This suggests that Fischer 344 rat macrophages are capable of containing the replication of these subsp. at least in part by the induction of NO. Thus, suppression of NO production resulted in a bacterial replication profile which more closely resembled that of hMDMs. The *in vitro* intracellular replication profile of the *Francisella* strains tested corresponded to *in vivo* susceptibility of Fischer 344 rats to pulmonary challenge. Importantly, Fischer 344 rats were markedly resistant to both LVS and *subsp. novicida*, while mice are highly sensitive to these two avirulent human strains [11], [13]. While both subsp. *tularensis* and *holarctica* causes serious disease in humans, the former is associated with much higher risk of mortality, which also is reflected in the rat model by the lower pulmonary LD_50_ dose of subsp. *tularensis*. Although there was minimal replication of subsp. *tularensis* within BMDM, there was clear evidence for robust bacterial replication in primary hepatocytes from the liver, a known site for disseminated bacterial replication [10], [24]. However, this does not rule out the ability of subsp. *tularensis* to replicate in primary macrophages isolated from the lung (alveolar macrophages) or peritoneal cavity (ongoing studies).

The establishment of the rat model of pulmonary tularemia is especially attractive for the screening of defined vaccine candidates, given the similar sensitivity of the rat to the various *F. tularensis* strains as humans. Mice have proven to be difficult to protect against subsp. *tularensis* pulmonary challenges, and this may be due to the extreme sensitivity of the mouse to all *F. tularensis* strains, even those that cause little to no disease in humans, such as subsp. *novicida* [9], [10], [11], [13]. Because mouse infections with attenuated subsp. *novicida* strains provide little to no protection against subsequent subsp. *tularensis* challenge, this subspecies has been ignored largely as a potential tularemia vaccine platform. However, in this study we have shown that subsp. *novicida* vaccinated rats exhibited a polarized Th1-type immune response and maintained a high degree of protection to a lethal pulmonary challenge with subsp. *tularensis*. These findings suggest that subsp. *novicida* should be reconsidered as a potential tularemia vaccine platform. In this regard, subsp. *novicida* has several distinct advantages, including the inherent low virulence this subspecies exhibits in humans, the ease of genetic manipulation [12], [39], [40], and the lack of select agent restrictions placed on this BSL-2 organism. Thus, the rat model may be a suitable system for the comprehensive screening of defined subsp. *novicida* vaccine strains for protective efficacy against subsp. *tularensis* pulmonary challenge. Our results extend those of Wu *et. al*. [24] who showed that LVS vaccination of the rat provides a large window (at least 4 log_10_) of protection against subsp. *tularensis* challenge. These studies provide evidence that the Fischer 344 rat may be an important animal model in the development of a licensed vaccine against human tularemia.

## Materials and Methods

### Ethics Statement

All animal experiments were performed in compliance with the Animal Welfare Act, the U.S. Public Health Service Policy on Humane Care and Use of Laboratory Animals and “Guide for the Care and Use of Laboratory Animals” published by the National Research Council. Animal work was done in accordance with the guidelines set forth by the University of Texas at San Antonio Institutional Animal Care and Use Committee (IACUC) under approved protocol number MU031-11/11A0.

### Animals

Four to six week old Fisher 344 rats and 4 week old BALB/c mice were purchased from the National Cancer Institute (Bethesda, Maryland). Animals were housed at the University of Texas at San Antonio animal facility and given food and water *ad libitum*.

### Bacteria

*Francisella tularensis* (*F.t*.) subsp. *novicida* strain U112 was provided by Dr. Francis Nano at The University of Victoria, Canada. *F.t.* subsp. *holarctica* LVS (Lot 703-0303-016) was obtained from Dr. Rick Lyons at The University of New Mexico. *F.t.* subsp. *holarctica* strain OR96-0246 and *F.t.* subsp. *tularensis* strain SCHU S4 were obtained from the Centers for Disease Control. All strains were grown from original stocks in tryptic soy broth (TSB) or on tryptic soy agar (TSA), each supplemented with 0.1% (w/v) L-cysteine. Bacterial titers were determined by plating of serial dilutions on TSA + cysteine.

### Intratracheal challenges

Pulmonary challenges were performed by intratracheal inoculation as described previously [24], [41]. Briefly, rats were anesthetized in 5% isoflurane with oxygen at 2 liters per min using a rodent anesthesia machine (Harvard Apparatus, Holliston, MA) and then placed dorsally on a surgical platform (Alpha Lab Supply; Albuquerque, NM). Using a laryngeoscope blade (Penn-Century, Inc; Philadelphia, PA) to expose the trachea and secure the tongue, a 20 gauge plastic catheter was inserted into the trachea. A blunt-end needle was then inserted into the plastic sheath and 100 µl of inoculum was delivered, followed by 300 µl of air to ensure the entire inoculum reached the lungs. The catheter was then removed and the rats were allowed to awaken before being returned to their cages, followed by daily monitoring for morbidity and mortality.

### Generation of bone marrow derived macrophages

Four to five week old rats and mice were euthanized by CO_2_ asphyxiation. Femurs were removed aseptically and washed in Dulbecco's Modified Eagles Medium (DMEM; Mediatech, Fairfax, VA) with 10% (v/v) fetal bovine serum (FBS; HyClone, Logan, UT) supplemented with L-glutamine (2 mM) and penicillin (100 U/ml)/streptomycin (100 µg/ml) (D10). The ends of femurs were removed and marrow was flushed from the bones with 10 ml of D10 and then centrifuged at 1200 RPM for 5 min at 4°C. The supernatant was discarded and the marrow was washed two more times in D10, resuspended in conditioned media (DMEM with 20% (v/v) FBS, L-glutamine, penicillin/streptomycin and 10% (v/v) supernatant from the L929 hybridoma cell line [42]), placed in a 75 cm^2^ tissue culture flasks and incubated at 37°C with 5% CO_2_. After 24 hr, non-adherent cells were removed and placed in a second flask to recover additional cells. Media was replaced every two days while cells differentiated, with the last two medium changes using conditioned medium without penicillin/streptomycin, and the resulting cells were utilized 8 days after collection. Cells were stained with mouse anti-rat CD11b AlexaFluor 647 (AbD Serotec, Raleigh, NC) or rat anti-mouse CD11b allophycocyanin (APC) (BD Biosciences) and determined to be >92% pure macrophages by flow cytometry.

### Fluorescent bead assay

Rat and mouse bone marrow derived macrophages (BMDM) were suspended in DMEM, seeded in 6 well plates at a density of 1×10^6^ cells per well and allowed to adhere. Yellow-green fluorescent (505/515 wavelength) carboxylate-modified microspheres, (Invitrogen, Carlsbad, CA) 1.0 µm in diameter, were added to the cells at a density of either 10 or 100 beads per cell and incubated for 2 hr to allow for phagocytosis of the beads. Remaining extracellular beads were then washed off, and cells were collected into tubes, stained with either mouse anti-rat CDllb AF647 or rat anti-mouse CD11b APC, and analyzed by flow cytometry.

### Phagocytosis assays

BMDM from Fisher 344 rats were suspended in D10, seeded into 96-well plates at a density of 2×10^5^ per well, and allowed to adhere. Macrophages were infected (10 and 100 multiplicity of infection [MOI]) with *Francisella* strains for 2 hr, washed two times with D10, gentamycin treated (20 µg/ml) for 1 hr, washed three times with D10, and then incubated in D10 for up to 72 hr. At indicated time points (3, 24, 48, 72 h) after challenge, supernatants were removed and stored at −80°C for cytokine analysis. Cells were then lysed with 0.2% deoxycholate and serial dilutions of the lysates were plated on TSA + cysteine to determine intracellular bacterial counts. In separate experiments, cells were lysed immediately following the 2 hr infection period and serial dilutions of both cell lysates and supernatants were plated to determine bacterial numbers. In other experiments, at the time of challenge, naïve rat serum was added as an opsonin, or 1 mM N-methyl-L-arginine acetate salt (Sigma Aldrich, St Louis, MO) as a nitric oxide inhibitor [43].

### Analysis of culture supernatants

NO production in overnight cultures was measured as nitrite concentration in culture supernatants by the Greiss assay essentially as described [44]. Supernatants from *in vitro* phagocytosis assays were analyzed by ELISA for the presence of TNFα according to manufacturer's instructions (BD Biosciences).

### Hepatoctye isolation and intracellular replication assays

Fischer 344 rats were euthanized, livers harvested, and hepatocytes isolated by a modification of the method of Seglen et. al. [45]. Briefly, the liver was perfused with 100 ml of Hank's balanced salt solution (w/o calcium and magnesium) (HBSS; Mediatech), followed by 100 ml of HBSS plus 5 mM calcium and 0.5 mg/ml Type IV collagenase (Sigma Aldrich) and subsequent removal of the liver into a sterile Petri dish. The tissue was gently disrupted to release hepatocytes and the cells were centrifuged at 29 X G for 3 min at 4°C and washed two times in HBSS. Hepatocytes were seeded in D10 at a density of 2×10^5^ per well in 96 well tissue culture plates and incubated for 2 hr at 37°C to allow the cells to adhere. The rat hepatocytes were then infected at an MOI of 10 or 100 with subsp. *holarctica* or *tularensis* and assayed for intracellular bacterial replication.

### Validation of vaccination efficacy of subsp. *novicida*

Rats were challenged intratracheally with either 10^2^ or 10^5^ CFU of subsp. *novicida* in PBS or mock-challenged with PBS alone and rested for 30 days. Rats were then given a secondary intratracheal challenge of 10^4^ CFU of subsp. *tularensis*, a dose which was previously determined [24] to be lethal to naïve rats, and monitored daily for morbidity and mortality. To determine cellular responses to subsp. *novicida* challenge, some rats were euthanized 14 days after challenge with 10^5^ CFU of the organism, and cervical lymph nodes collected. Single cell suspensions were prepared and cultured (1×10^6^ cells/well) for 72 hr in DMEM supplemented with 10% (v/v) FBS±10^5^ CFU of UV-inactivated subsp. *novicida*. Bacteria were inactivated by exposure to a 30 W short wavelength UV light source for 15 min at a distance of 15 cm, and the inactivation confirmed by absence of bacterial growth on TSA+cysteine plates. Some cells also were cultured with the unrelated antigen hen egg lysozyme (HEL). Culture supernatants were harvested for IFN-γ analysis by ELISA per manufacturer's instructions (eBioscience, San Diego, CA). To determine humoral responses to subsp. *novicida* challenge, rats were bled and sera prepared 30 days after intratracheal challenge with 10^5^ CFU. Briefly, microtiter plates were coated overnight with 10^6^ CFU of UV-inactivated subsp. *novicida* in sodium bicarbonate buffer (pH 9.5), washed with PBS containing 0.3% Brij-35 (Sigma), and blocked for 2 hr at room temperature with PBS containing 5% FBS and 0.1% Brij-35 as described previously [11]. Serial dilutions of serum were added to wells and incubated at room temperature for 2 hr. The plates were then washed and incubated for an additional 2 hr with goat anti-rat total Ig, IgG1 and IgG2a conjugated to horseradish peroxidase (Southern Biotechnology Associates, Birmingham, AL). After incubation, the plates were washed and teramethylbenzidine substrate (BD Biosciences, San Diego, CA) was added for color development. The reciprocal serum dilutions corresponding to 50% maximal binding were used to obtain titers. No binding of immune serum was detected in plates coated with the unrelated antigen HEL.

### Statistics

SigmaStat (Systat Software Inc., San Jose, CA) was used to perform tests of significance. Student's *t* test was used to determine differences in intracellular replication, cytokine and antibody production. Statistical analysis for survival experiments was performed using the Kaplan-Meier test. All data are reported as mean ± standard error from each experimental animal group and are representative of at least two independent experiments.
